# Nitric oxide pathway-mediated relaxant effect of aqueous sesame leaves extract (*Sesamum radiatum *Schum. & Thonn.) in the guinea-pig isolated aorta smooth muscle

**DOI:** 10.1186/1472-6882-8-23

**Published:** 2008-05-27

**Authors:** André B Konan, Jacques Y Datté, Paul A Yapo

**Affiliations:** 1 Laboratoire de Nutrition et Pharmacologie, Département BA-PA, UFR-Biosciences, Cocody University, Abidjan 22 BP 582 Abidjan 22, Côte d'Ivoire; 2 Laboratoire de Physiologie Animale, Pharmacologie et Phytothérapie Abobo-Adjamé University, Abidjan, Côte-d'Ivoire

## Abstract

**Background:**

*Sesamum radiatum *Schum. & Thonn. (Pedaliaceae) is an annual herbaceous plant, which belongs to the family Pedaliaceae and genus *Sesamum*. Sesame is used in traditional medicine in Africa and Asia for many diseases treatment. Sesame plant especially the leaves, seed and oil are consumed locally as a staple food by subsistence farmers. The study analyses the relaxation induced by the aqueous extract of leaves from sesame (ESera), compared with those of acetylcholine (ACh) in the guinea-pig aortic preparations (GPAPs), in order to confirm the use in traditional medicine for cardiovascular diseases.

**Methods:**

The longitudinal strips of aorta of animals were rapidly removed from animals. The aorta was immediately placed in a Mac Ewen solution. Experiments were performed in preparations with intact endothelium as well as in *aortae *where the endothelium had been removed. The preparations were suspended between two L-shaped stainless steel hooks in a 10 ml organ bath with Mac Ewen solution. The isometric contractile force of the aorta strips of guinea-pig were recorded by using a strain gauge. All both drugs caused concentration-dependent relaxations responses.

**Results:**

The aqueous extract of leaves from sesame ESera (1 × 10^-7 ^– 0.1 μg/ml) caused a graded relaxation in GPAPs with intact endothelium, with a EC_50_-value of 1 × 10^-4 ^μg/ml. The same effect was observed with ACh (7 × 10^-2 ^nM – 7 × 10^-1 ^μM), which caused relaxation in a concentration-dependent manner. The relaxation in response to ESera and, like that to ACh in GPAPs without endothelium, was fully abolished. Destruction of the endothelium or incubation with the nitric oxyde synthase inhibitor (L-NNA) significantly enhanced the inhibition of the relaxation response to ESera. Moreover, all concentrations induced vasoconstrictions. However, L-NNA produced a significant displacement to the right (about 65-fold) of the relaxation response to ESera. Similar results were obtained with ACh. Both diclofenac and tetra-ethyl-ammonium (TEA) pretreatment of GPAPs induced a suppression of the relaxation caused by ESera, and produced a very significant rightward shifts of the CRC (16-fold) for diclofenac and increase the Emax. In contract, the relaxation caused by ACh was not significantly affected by diclofenac or by TEA.

**Conclusion:**

Thus, the present results indicate clearly that the nitric oxide largely contribute to the relaxation effect of Esera and of ACh in GPAPs. In addition, their contractile effects are also mediated by cyclooxygenase activation, and probably the K+ channels involvement, that confirm the use of various preparations of Esera for the treatments of cardiovascular diseases.

## Background

*Sesamum radiatum *Schum. & Thonn. (Pedaliaceae), pantotropical plant was used in traditional Medicine to facilitate the delivery in pregnant women [1,2]. Our first investigation with the action of ESera in the cardiovascular system of mammalians showed that ESera as well as acetylcholine, induced arterial hypotension resulting from cardiodepression and vasorelaxation of rabbits and rats [3]. Indeed, Furchgott et al (1980) [4] showed the mechanism of acetylcholine on the vascular smooth muscle. The vasodilatation induced by acetylcholine depended on the functional integrity of the endothelium and implied an endothelial hyperpolarising factor (EDHF) and a release of nitric oxide (NO). For these authors, the releasing effects of acetylcholine were extended to other natural biological substances or from synthesis [5,6].

In view of these findings, the current study pursued three aims. The first goal is to explore whether ESera is able to inhibit GPAPs contractile activity. The second aim was to compare ESera and ACh in their potency and efficacy to relax the contractile force in the presence of nitric oxide. The third aim of the study was to explore whether any effect ESera on aorta smooth muscle is exclusively due to activation of cyclooxygenase pathway in relationship with nitric oxide (NO) or whether there is also cholinoceptor-independent component. Finally, the present study is an approach to elucidate the mechanism underlying ESera's action in the vasoralaxation observed in GPAPs.

## Methods

### Plant

*Sesamum radiatum *(Schum.) Thonn. (Pedaliaceae) was collected in October 2005 from farms specialized in growing plants for scientific or medical purposes. The leaves of *Sesamum radiatum *were verified to be identical samples at the specimen herbarium of the Centre National Floristique de Côte d'Ivoire at Cocody University in Abidjan. Voucher specimen were preserved and catalogued in the same herbarium (Centre National Floristique). This pantropical plant was authenticated by a Botanic expert, Prof. Aké-Assi Laurent of Centre National Floristique, UFR-Biosciences, University of Cocody, in Abidjan, Côte d'Ivoire).

### Phytochemical screening

The extract and its fractions were tested by Lieberman Bouchard, Ferric chloride, cyanidine stiasny and, Valser-Meyer and Dragendorff Tests to determine the presence of strerols, phenolic compounds, flavonoids, Tannin and alkaloids, respectively.

### Preparation of extract of sesame leaves

*Sesamum radiatum *leaves were stored in cellophane bags. The leaves collected were dried at room temperature (Temperature: 27 ± 3°C). The powdered leaves (100 g) were first macerated for 24 hours in *n*-hexane to remove chlorophyll and other hexanosolubles substances. The residue was dried and extracted by vigorously shaking in bi-distilled water for 24 hours. After 2-h extraction and filtration, the filtrate was concentrated, by evaporation of the solvent. The drug (ESera) obtained was stored at 4°C until use.

### Animals

Guinea-pigs (*Cavia porcellus*), of both sexes weighing between 350 g and 400 g, were obtained from the Animal House of the Laboratory of Nutrition and Pharmacology of UFR-Biosciences at Cocody university in Abidjan (Ivory Coast). The guinea-pigs were housed in a constant temperature rooms with a light/dark cycle of 14/10 hours. The animals were fed and given water *ad libitum *until they were used.

### Preparation of the guinea-pig aorta

After sacrifice of animals, by cervical dislocation, the aorta was rapidly removed, and after being freed from connective tissue, each aorta was cut into three longitudinal strips (6–7 mm). The aorta was immediately placed in a Mac Ewen solution (at room temperature) of the following composition [(mM): NaCl: 130; KCl: 2.5; CaCl_2_: 2; NaH_2_PO_4_: 1.18; NaHCO_3_: 11.9; MgCl_2_: 0.24; glucose: 2.2 gassed with 95% O2 + 5% CO2].

Since it has been shown that endothelium can inhibit the vasorelaxator effect of acetylcholine in aorta preparations [7,8], experiments were performed in preparations with intact endothelium as well as in aortae where the endothelium had been removed by gentle rubbing of the intimae with a wooden rod to avoid the inhibitory role of functional endothelium to acetylcholine and Esera. Each strip of aorta was cut into 6–7 mm length. The preparations were suspended between two L-shaped stainless steel hooks in a 10 ml organ bath with Mac Ewen solution at 37°C (pH = 7.4). Each preparation was connected by a silk thread to a force transducer FT30 (Hugo Sachs electronic, Freiburg, Germany) and the isometric force was recorded by a pen-recorder Rikadenki (Freiburg, Germany) on paper enrolled at a speed of 2.5 mm/min. A resting tension of 1 g was maintained, this setting allowed for the optimum observation of maximal contractile response to drugs.

### Experimental protocol

After the equilibration period of 60 min, tissues were exposed to ESera, which was added to the bath by means of cumulative methods [9] and to depolarizing potassium solution to test the viability. In most cases, a maximal contractile response ranging from 0.8–1.1 g could be induced. The preparations with a response below 0.6 g were considered insufficiently viable and discarded.

The potassium solution (80 mM K^+^) had the same composition as the Mac ewen buffer used, except that NaCl had been completely replaced by an equimolar amount of KCl. Once the contraction had reached a plateau, the preparations were washed with Mac Ewen solution four times and left a further 40 min equilibration period at a re-adjusted tension of 1 g.

To avoid tachyphylaxis, only a single cumulative concentration-response curve (CRC) for drugs was obtained in each preparation. Appropriate controls were run at the same time in different strips obtained from the aorta.

At the end of each experiment, after the drugs had been washed-out with Mac Ewen solution four times, acetylcholine (1 μM) was added when the maximal response to noradrenaline (0. 4 μM) had been obtained, in order to assess the presence or absence of functional endothelium. A rapid and marked reduction of noradrenaline induced tone was taken evidence that a significant amount of functional endothelium was present. The absence of relaxant response was taken as indicative of the disappearance of functional endothelium [8,10].

### Influence of the endothelium on the effects of Esera

After the equilibration period of at least 60 min, preparations with or without endothelium were exposed to ESera, which was added to the bath by means of a cumulative method [9], cumulative CRCs for Esera (1 × 10^-7^–0.1 μg/ml) or ACh (1 × 10^-5 ^– 0.1 μg/ml) were constructed in both endothelium-intact and endothelium-denuded aortic preparations of the same aorta. The drugs were then washed out with Mac Ewen solution four times. Subsequently, noradrenaline (1 μM) -induced contractions were imposed and ACh (1 μM) was added to test whether functional endothelium was present.

### Influence of the nitric oxide (NO) synthesis inhibitor on the effects of Esera

After equilibration, GPAP were exposed to a nitric oxide (NO) synthesis inhibitor; N^ω^-Nitro-L-Arginine (L-NNA) at a concentration of 50 nM for 30 min. Cumulative CRCs of ESera were obtained in the presence of the nitric oxide inhibitor.

### Influence of the cyclooxygenase inhibitor on the effects of Esera

After equilibration, the preparations were exposed to a cyclooxygenase inhibitor diclofenac at a concentration of 30 nM for 30 min. Cumulative CRCs of Esera were obtained in the presence of the cyclooxygenase inhibitor.

### Influence of the non selective potassic channels blocker on the effects of Esera

After equilibration, the preparations were exposed to non selective potassium channels blocker the Tetra-ethyl-ammonium (TEA) at a concentration of 50 nM for 30 min. Cumulative CRCs of Esera were obtained in the presence of TEA.

### Chemicals used

The acetylcholine and the N^ω^-Nitro-L-Arginine were purchased from Sigma Company (St Louis, Mo, USA). Diclofenac and Tetra-ethyl-ammonium were provided from Sigma-Aldrich (Switzerland). All drugs were dissolved in saline.

### Statistics analysis

Responses were expressed as absolute changes in mg of tension. Data are expressed as means ± SEM obtained from *n *separate experiments. Statistical analysis of the results was determined by using the unpaired Student's *t*-test. *p *< 0.05 or less was considered as indicative of significance. The EC_50 _(i.e. the concentration of ESera or drug causing half maximum response) values were determined from individual experiments for the complete agonist concentration-response curves by figures carried out respectively using the software GraphPad Prism (San Diego, California, USA). The EC_50 _values are reported as geometric means accompanied by their respective 95% confidence limits. All other reported results are means ± s.e.m.

## Results

### Phytochemical screening

Phytochemical study that the sesame plant is rich in phenolic compounds (phenols, lignans and flavenoids). Sterols were also found in aqueous extract and its fractions.

### Extract of *Sesamum radiatum *Schum. & Thonn. (ESera) induced contractions in endothelium-denuded guinea-pig aortic preparations

In control conditions, regular contractile activity of the isolate aorta was recorded which were constant in all experiments and were not influenced by the addition of vehicle (Mac Ewen's solution (original tracing not shown).

**Figure 1:**
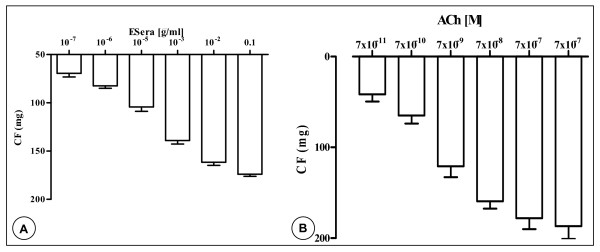
Mean relaxant concentration-response curve for the effect of the extract of *Sesamum radiatum *Schum. & Thonn (**A**) and for acetylcholine (ACh, **B**) in the guinea-pig isolated aorta preparations with endothelium. Data are shown as mean ± s.e.m., expressed as the absolute values of percentage of the maximal response of n experiments (n = 4–6, p < 0.05).

In endothelium-intact GPAPs, ESera applied in a concentrations range from 1 × 10^-7 ^to 10^-1 ^μg/ml caused relaxation of aorta preparations, in a concentration-dependent manner. The calculated means EC50 (95 % confidence limits) and maximum relaxations for this effect were: 1 × 10^-4 ^μg/ml. Concentrations of 1 × 10^-7 ^μg/ml and of 10^-1 ^μg/ml induced relaxations of 69.6 ± 9 mg and of 174.2 ± 5,8 mg, respectively. Similar observations were obtained in the presence of acetylcholine. Both ESera and acetylcholine on the relaxation of GPAPs was depicted on Fig. 1A &1B.

Figure 1B illustrates the effect of acetylcholine (ACh) on the activity of GPAPs. ACh (7 × 10^-2 ^nM – 7 × 10^-1 ^μM) caused relaxation, in a concentration-response manner (EC_50_-value = 5.5 nM). ACh at concentrations of 7 × 10^-2 ^nM and 0.7 μM induced relaxation of endothelium-intact GPAPs for 41.7 ± 8 mg and for 187 ± 12 mg, respectively.

**Figure 2:**
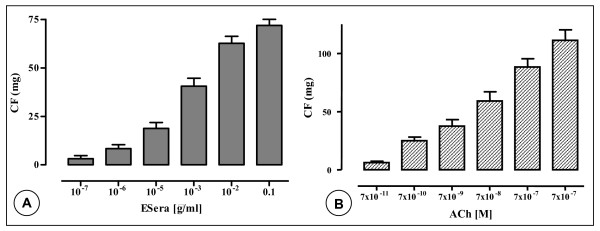
Mean relaxant concentration-response curve for the effect of the extract of *Sesamum radiatum *Schum. & Thonn (**A**) and of acetylcholine ACh (**B**) in the guinea-pig isolated aorta preparations without endothelium. Data are shown as mean ± s.e.m., expressed as the absolute values of percentage of the maximal response of 4 experiments (p < 0.05).

In endothelium-denuded GPAPs, ESera in the same concentrations range induced contractions of isolated *aortae*. Concentration of ESera of 0.1 μg/ml induced a contractile force of 90.6 ± 6 mg (Fig. 2A). Contractions were obtained with ACh in the same preparations, which induced concentration-dependently contractions of endothelium-denuded GPAPs. Concentration of 7 × 10^-2 ^nM induced a contractile force of 111.9 ± 9 mg (Fig. 2B).

### Inhibitory effects of L-N ^omega^-Nitro- Arginine on ESera-induced vasorelaxation in guinea-pig aorta strips

Our previous experiments showed that ESera and ACh induced vasorelaxation of endothelium-intact guinea-pig aorta smooth muscle. In order to justify the involvement of the possible modulation by the endothelium of the contractile responses to ESera, L-NNA was used.

**Figure 3:**
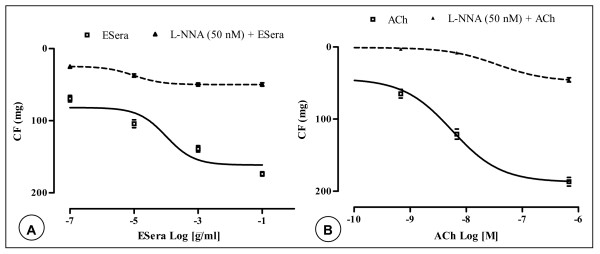
Mean relaxant concentration-response curve for the extract of *Sesamum radiatum *Schum. & Thonn in endothelium-intact (**A**) or in endothelium-denuded (**B**) guinea-pig isolated aorta preparations in the absence (□) and in the presence (▲) of L-NNA. Data are shown as mean ± s.e.m., expressed as the absolute values of percentage of the maximal response of n experiments (n = 4–6, p < 0.05).

L-NNA (50 nM) shifted the CRCs for ESera concentration-dependently in endothelium-intact GPAPs to the right (EC_50_-value = 7.4 × 10^-4 ^μg/ml). However, the E-max-value was greatly attenuated of 71%. High concentration of ESera (0.1 μg/ml) caused a significant reduction of the maximum relaxation (174.2 ± 5.8 mg) by the nitric oxide synthase inhibitor, to 50 ± 4 mg (Fig. 3A). Similar results were obtained with ACh. Indeed, L-NNA (50 nM) caused a rightward shift of the CRCs of ACh in endothelium-intact GPAPs (EC50-value = 36 nM). ACh (0.7 μM) induced a relaxant effect of 187 ± 12 mg (Figure 3B) and a decrease of this effect induced by ACh to 45 ± 6 mg (an inhibition of relaxation of 75 %).

### Inhibitory effects of diclofenac or tetra-ethyl-ammonium on ESera-induced vasorelaxation in guinea-pig aorta strips

Previous studies of the effects of ESera were carried out on the cardiac muscle of rat in the presence of the atropine, antagonist of the muscarinic cholinoceptors. This study showed a partial cholinergic antagonist action of ESera [2].

The relaxations induced by Esera in GPAPs with endothelium were antagonized in a concentration-dependent and reversible manner by both diclofenac (cyclooxygenase inhibitor) and TEA (non-selective K^+ ^channel blocker).

**Figure 4:**
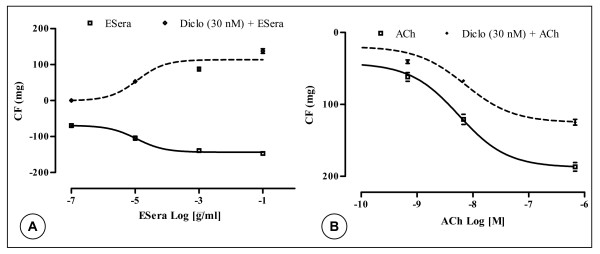
Mean relaxant concentration-response curve for the effect of extract of *Sesamum radiatum *Schum. & Thonn (**A**) and of acetylcholine (**B**) in endothelium-intact guinea-pig isolated aorta preparations in the absence (□) and in the presence (◊) of diclofenac. Data are shown as mean ± s.e.m., expressed as the absolute values of percentage of the maximal response of n experiments (n = 6, p < 0.05).

The diclofenac (30 nM) pre-treatment of endothelium-intact GPAPs caused ESera-induced vasocontriction. Diclofenac (30 nM) shifted the CRCs for ESera concentration-dependently in endothelium-intact GPAPs to the right (EC_50_-value = 1.2 × 10^-5 ^μg/ml). The contractile force was 90 ± 6 mg, when ESera applied at a concentration of 0.1 μg/ml (Figure 4A). However, the same pre-treatment with diclofenac unaffected the ACh-induced relaxation of endothelium-intact GPAPs (EC_50 _= 7.2 nM). The value of the vasorelaxation was 125 ± 7.8 mg at a ACh's concentration of 0.7 μM. In contract, diclofenac did not significantly affect the relaxation induced by ACh.

In endothelium-intact GPAPs ESera-induced relaxation was significantly affected by the potassium channels blocker TEA (50 nM), but was potentiated by the cyclooxygenase inhibitor, diclofenac (30 nM).

**Figure 5:**
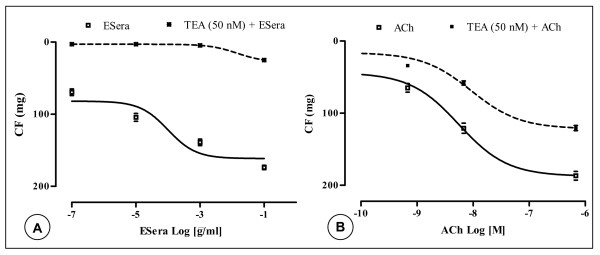
Mean relaxant concentration-response curve for the effect of extract of *Sesamum radiatum *Schum. & Thonn (**A**) and of acetylcholine **(B)** in endothelium-intact guinea-pig isolated aorta preparations in the absence (□) and in the presence (●) of TEA. Data are shown as mean ± s.e.m., expressed as the absolute values of percentage of the maximal response of n experiments (n = 6, p < 0.05).

In endothelium-intact GPAPs, TEA at the same concentration inhibited totally the vasodilatory action to ESera. The CRC for ESera was shifted to the right (EC_50_-value = 1.2 × 10^-2 ^μg/ml, Fig. 5A). In contrast, TEA caused slightly a rightward shift of the CRCs of ACh-induced relaxation in a concentration-response manner (EC_50_-value = 9.2 nM, Fig. 5B). Interestingly, neither TEA nor diclofenac interfered significantly with the relaxation produced by ACh.

## Discussion

Herbal preparations are used since ancient times to maintain health. Also, herbal preparations, if taken in appropriate dose, can lead to a better option for curing various aliment [11,12].

The results of present study have demonstrated that ESera, besides inducing concentration-dependent relaxation in endothelium-intact GPAPs, at the same concentrations produces concentration-dependent contraction in either endothelium-denuded GPAPs or in the presence of nitric oxide synthesis inhibitor. Esera-induced relaxations in GPAPs with endothelium-intact are counteracted by endothelium.

Leaves of *S. radiatum *are used traditionally by patients in West Africa and are taken as water decoction [13]. Due to this reason the leaf juice of the plant was evaluated and the data also confirmed the traditional indications. Early investigations [2,14] on the antihypertensive properties of the extracts of *S. radiatum *leaves by other authors also substantiate the results of our studies in Rabbits [3].

It seems likely that the endothelium-induced inhibition of ESera-effects is mediated by the endogenous vasodilator endothelium derived relaxing factor (EDRF) [10,11]. EDRF is assumed to be identical with NO or a closely related compound and vascular endothelial cells are known to synthesize NO [16,17].

The releasing mediators are, for the majority, intrinsically diminishing vascular smooth muscle tone. The endothelial cells synthesize, according to stimuli, of the molecules to the vasorelaxant character such NO, protaglandins (PGE_2_) with are releasing endothelial factors (EDRF) and the endothelial hyperpolarize factor (EDHF) [18-22].

Our findings are the first demonstration of endothelium-intact contractions to ESera in GPAPs.

Sesame had been shown to possess multiple health benefits, both alone and in synergistic combination with others compounds. By influencing pharmacological processes in the body, sesame and its lignans promise to help reduce risk for many of today's most common diseases, including heart disease, obesity, arterial hypertension and inflammatory disorders [23-31].

On the other hand, the TEA and the diclofenac differently affect the contractile activity induced either by ACh or by ESera. In separate experiments, after the first CRC of ESera had been obtained in endothelium-intact, 30 nM diclofenac or 50 nM TEA were added and then the second CRC was contracted. It was fund that the maximal responses of second curves were increased in the presence of either antagonist. Diclofenac slightly shifted the second curves of ACh to the right, whereas it had no significant effect on the curves compound. These data, taken together with other results in this study, suggest that the contractile response to ESera is mediated by cyclooxygenase-receptors. Modulating factors which influence these parameters can be released from endothelium or from smooth muscle cells.

The prostaglandins as well as indometacin, a powerful antagonist of the cyclooxygenase did not affect relaxation in response to ACh [32,33].

Destruction of the endothelium or inhibition of NO synthesis significantly enhanced the response to ESera in this preparation. Moreover, similar results were obtained with cyclooxygenase-inhibition or K^+ ^channels blocker were obtained with Chen *et al*. [34] and Niu *et al*. [35] suggest that the endothelium-denuded affect by NO via K^+ ^channels involvement cyclooxygenase pathways.

Our study indicated a significant myorelaxant and supports the traditional use of fresh leaves by Ivorian physicians.

In conclusion, on the basis of beneficial effect of ESera in the literature and our own results of the experiments in the extract of the same species ESera induces a decrease of vascular tone. Once found ESera may be incorporated as antihypertensive agent for the improvement of the patients suffering from cardiovascular diseases.

Chemical and pharmacological studies are now in progress to isolate and to characterize the constituents responsible for such effects, and also to investigate in more detail the mechanisms underlying the relaxant action of the active principle (s) of ESera in GPAPs.

## Competing interests

The authors declare that they have no competing interests.

## Authors' contributions

All authors contributed equally in the study.

## Pre-publication history

The pre-publication history for this paper can be accessed here:


